# Reflections on the regulatory field covering the development of paediatric medicinal products: a brief overview of current status and challenges

**DOI:** 10.3389/fphar.2024.1375988

**Published:** 2024-06-03

**Authors:** Birka Lehmann

**Affiliations:** Drug Regulatory Affairs, Pharmacy Section, Rheinische Friedrich-Wilhelms-Universität Bonn, Bonn, Germany

**Keywords:** medicinal products, paediatric population, clinical trials, medicinal products approval, concepts

## Abstract

Many medicinal products are initially developed and tested in adults, and often, only a limited amount of data on their safety and efficacy in children exist. Consequently, paediatric healthcare providers sometimes need to make informed decisions about using medicinal products in children based on available fragmentary data. Ethical guidelines emphasise the importance of protecting children and ensuring they receive safe and effective medical treatments. Paediatric clinical trials should be conducted to provide evidence-based care with specific attention to minimise risks to children. This highlights the dilemma of finding a balance between protecting children and adolescents (and avoiding unnecessary clinical trials) and obtaining reliable, robust and justified data to treat them adequately and not in an off-label manner with unknown risks. For years, paediatricians maintained that children and adolescents are not treated based on up-to-date scientific knowledge and justification. The slogan “children are not small adults” summarised the concerns in a catch phrase. Different stakeholders have taken a variety of actions to address this concern.

## 1 Introduction

On a global level, the International Council for Harmonisation of Technical Requirements for Pharmaceuticals for Human Use (ICH), bringing together the regulatory authorities and pharmaceutical industry, developed and finalised the ICH guideline E 11 Guideline on the Clinical Investigation of Medicinal Products in the Pediatric Population in 2000 (International Council of Harmonisation et al., 2024a). Governments recognised the necessity for legal provisions to support the development of medicinal products for children and adolescents. The United States (US) (FDA U.S. Food and Drug Administration, 2018) and the European Union (EU) (European Commission, 2018) have implemented legal requirements mandatory for new medicinal products and optional for known medicinal products intended for paediatric use. Several other countries have followed this approach to take special care for the treatment of children and adolescents with medicinal products, which includes Canada (Government of Canada Canada, 2024), Australia (Australian Government Department of Health and Aged Care, 2020), Switzerland (Swissmedic, Switzerland Swiss Medic Medicines for Children, 2014), and Japan (PMDA, 2023b). Other countries, like Brazil, are monitoring the situation carefully, following the progress in the European Union and the United States. These new legal requirements had a tremendous influence on the development of (new) medicinal products and appropriate treatment options for the paediatric population. The complexity of the development of a paediatric medicinal product has to be taken into account. For a new medicinal product, pharmaceutical companies must adapt and extendtheir development programme to fulfil the legal obligation to include the paediatricpopulation, but also to obtain any marketing authorisation for adults. The respective authorities should closely follow and accompany this by providing supporting guidelines, adequately updated, and adapted to advancements in paediatrics and medicinal product development. In the end, the intended benefit of these new approaches will only be reached by a combination of unavoidable clinical trials, duly agreed to by ethics committees and competent authorities, adequate paediatric formulations, and final approval based on risk-benefit evaluation and the supporting reliable information. All stakeholders are still on a learning curve and will profit from accompanying up-to-date training and education. There is a need for regulators to adjust legislation and regulations, including implementation guidance that considers ‘real life’ conditions, for the pharmaceutical industry to leave the ‘beaten track’, and for paediatricians to adapt the treatment options to scientific progress. Due to references coming from different sources, both ‘paediatric’ or ‘pediatric’ and ‘medicinal product’ or ‘drug’ are used in the overview.

## 2 Historical development

Like the gap between Scylla and Charybdis, a trough exists between thinking of new ways and traditional approaches.

Paediatricians have worked for decades to address this issue. On one hand is the ethical consideration to not conduct clinical trials with children; on the other hand is the dilemma of not knowing whether a medicinal product would work in children and what kind of adverse reaction might occur, taking into account the differences in physiological and psychological development, which are further complicated by questions of the correct dose and adequate formulation. The ‘extrapolation concept’, either by per-kg/bodyweight or body surface based on data from adults, is not always appropriate and has led to over- or underdosing and, not the least, to ‘off-label’ treatment.

Within the 1990s, the scientific community came to the conclusion that the risk ratio between involving children in clinical trials and the day-to-day treatment of children without sufficient justification of the medicine itself or the dose clearly demanded a science-based approach.

This was taken up by the governments of the United States and European Union and is followed by health authorities in other countries.

In parallel, WHO and ICH have taken the concerns on board by implementing the WHO Paediatric Regulatory Network and publishing the ICH E11 guideline: Clinical Investigation of Medicinal Products in the Pediatric Population, in 2000, which was updated in 2017 (International Council of Harmonisation et al., 2024a).

This review will focus on the development of paediatric legislation in the United States and European Union and will give brief information on other actions in individual countries and cooperation between national competent authorities in different countries.

## 3 Global approach

### 3.1 International council of harmonisation

With the agreement on the ICH guideline E11, one big step was taken by defining the paediatric population and an age classification with respect to the physical and psychological development of children.

In Section 2.5, the ICH guideline gives a perspective of an ‘Age Classification of Paediatric Patients’ concerning clinical trials (in this population) as follows:

Any classification of the paediatric population into age categories is arbitrary to some extent, but a classification such as the one provided by ICH gives a basis for thinking about study design in paediatric patients. Decisions on how to stratify studies and data by age need to take into consideration developmental biology and pharmacology. Thus, a flexible approach is necessary to ensure that studies reflect current knowledge of paediatric pharmacology. The identification of which ages to study should be medicinal product-specific and justified.

The following is one possible categorisation. There is considerable overlap in developmental (e.g., physical, cognitive, and psychosocial) issues across the age categories. Ages are defined in completed days, months, or years:

• Preterm newborn infants
• Term newborn infants (0–27 days)
• Infants and toddlers (28 days–23 months)
• Children (2–11 years)
• Adolescents (12–16–18 years (dependent on region)) (ICH International Council of Harmonisation, 2024).

This guideline should be read in accordance with the ICH guideline S11 (European Medicines Agency EMA and, 2020a) with Supplementary Table SA1: age-dependent development of human organ systems.

ICH states:

The classification system is not a binding system in respect to the planning and conducting of clinical trials in the paediatric population. The classification system has to be adapted to the challenges coming from the disease. For example, the development of a medicinal product for the treatment of asthma has to take into account the age in relation to outbreak of the disease (European Medicines Agency EMA, 2015).

## 4 Legislation in the United States and European Union

### 4.1 United States

The following highlights the main provisions of the relevant US legislation as published by the FDA:

With the Pediatric Labeling Rule in 1994 (US Food and Drug Administration, 2023), the FDA made a first attempt to improve the situation. This was followed by the implementation of the Best Pharmaceuticals for Children Act (BPCA) (FDA U.S. Food and Drug Administration, 2024a) in 2002 and the Pediatric Research Equity Act (PREA) (FDA U.S. Food and Drug Administration, 2024b) in 2003.

The Pediatric Labeling Rule required the inclusion of particulars in the package information on the medicinal product if information for the treatment of children is available and led to changes in the requirements for the development of drugs.

The BPCA provided incentives if a pharmaceutical company voluntarily conducts paediatric clinical trials in indications inside or outside the development for adults.

The PREA made it mandatory for new drugs and biologics to also develop a drug for children and adolescents, restricted to the indication developed for adults. Orphan medicinal products were exempted.

In 2017 with the Research to Accelerate Cures and Equity (RACE) for Children Act, the PREA exemption for orphan medicinal products was amended to also cover drugs intended for the treatment of adult cancers and directed at a molecular target substantially relevant to the growth or progression of a paediatric cancer.

The submission of an initial paediatric study plan (iPSP) is mandatory with the implementation of both acts. The final paediatric study plan (PSP) is the FDA-approved document for the development of a drug for children and adolescents.

### 4.2 European Union

In Europe, no harmonised approach in EU Member States existed regarding information on the treatment for children and adolescents in the Package Leaflet or Summary of Product Characteristics before the Paediatric Regulation came into force.

Member States have taken different approaches to this topic. In Germany, for instance, a contra-indication for children below 12 years of age was commonly listed.

In 2006, the European Regulation (EC) No 1901/2006 was adopted and came into force in 2007 (European Commission Regulation and EC, 2019).

The EU Regulation follows the concept of a mandatory development of the medicinal product for children or adolescents if the medicinal product is a ‘new’ one for the EU market (Art. 7 and 8) and a voluntary development if the medicinal product is already on the EU market (Art. 30). The paediatric-use marketing authorisation (PUMA) is a dedicated marketing authorisation covering the indication(s) and appropriate formulation(s) for medicines developed exclusively for use in the paediatric population. One particular aspect of the EU Regulation is the inclusion of orphan medicinal products. The intention was and is to close the gap of ‘unmet medical need’.


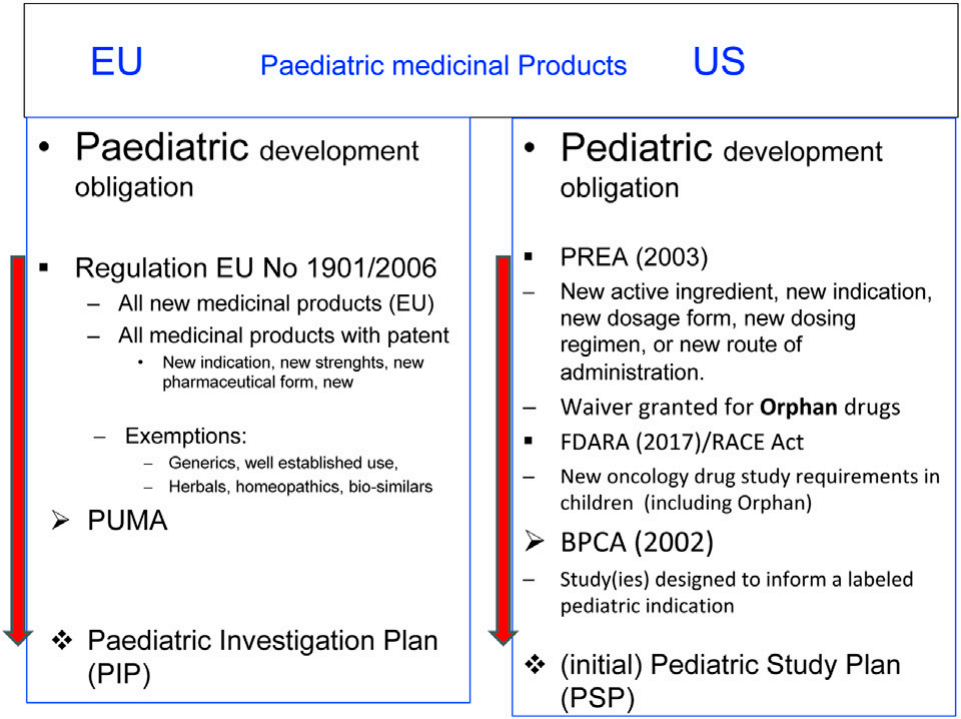


### 4.3 Administrative part

#### 4.3.1 US pediatric study plan (PSP)

The following highlights the main provisions, as published by the FDA:

The general rule of age classification is modified in the FDA guideline:

Neonates: birth through 27 days (corrected gestational age).

Infants: 28 days to 23 months.

Children: 2 years–11 years.

Adolescents: 12 years to younger than 17 years (FDA U.S. Food & Drug and European Medicines Agency EMA, 2020)

The FD&C Act requires that an iPSP includes the following (FDA U.S. Food & Drug and European Medicines Agency EMA, 2020):

(i) an outline of the pediatric study or studies that the sponsor plans to conduct (including, to the extent practicable, study objectives and design, age groups, relevant endpoints, and statistical approach);
(ii) any request for a deferral, partial waiver, or waiver. if applicable, along with any supporting information; and
(iii) other information specified in the regulations issued by the FDA.

All or some paediatric assessments, due to development, may be deferred if

• Marketing authorization is ready for approval in adults
• Additional safety or effectiveness data are needed before paediatric studies
• Other reasons.

The FDA has recently emphasized that an iPSP must be submitted with the request for deferral and include:

• Evidence that the studies are being conducted or will be and
• A timeline for completion of such studies.

Annual status reports detailing progress or evidence that studies will be conducted at the earliest possible time will be made public.

The development of a medicinal product for children may be waived under the following conditions:

If studies are “impossible or highly impracticable:”

• For example, small patient population* or geographically dispersed;
• Evidence of ineffectiveness or unsafe;
• No “meaningful therapeutic benefit” AND not likely to be used in a substantial number of patients. Under pediatric rule, a substantial number = 50,000 patients*;
• Inability to develop a paediatric formulation.

This has been modified by the RACE Act.

#### 4.3.2 EU paediatric investigation plan

The general rule of the ICH E11 guideline is modified in the EU recommendations as follows:

Preterm and term neonates from 0 to 27 days;

Infants (or toddlers) from 1 month to 23 months;

Children from 2 years to 11 years; and

Adolescents from 12 up to 18 years (EUROPEAN COMMISSION, 2014).

In the European Union, it is common practice to divide children’s age groups into children 2–6 years and school children 6–11 years (Adriana et al., 2011).

The communication of the commission, ‘Guideline on the format and content of applications for agreement or modification of a PIP and requests for waivers or deferrals and concerning the operation of the compliance check and on criteria for assessing significant studies’ gives detailed information regarding the content and supporting documents needed to apply for a PIP, the compliance check and assessing significance of studies (EUROPEAN COMMISSION, 2014).

Deferrals may be granted for specific conditions due to delaying development of the medicine for children until, for instance, there is enough information to demonstrate its effectiveness and safety in adults.

Even when studies are deferred, the PIP must include details of the paediatric studies and their timelines.

The deferral of a PIP is linked to the obligation to submit annual reports to the agency. These reports should provide an update on progress with paediatric studies in accordance with the decision of the agency agreeing to the PIP and granting a deferral (European Medicines Agency, 2006).

The development of a medicinal product for children and adolescents may be waived.

All medicinal products identified in Article 9 of the regulation 1910/2006., are exempted from the obligation.

The European Medicines Agency published a list of medicinal products that are exempted from the obligation to develop the medicinal product for the paediatric population due to their mode of action and a list of diseases in which it is assumed that the disease or condition will not appear in the paediatric population.

In addition to these class waivers, either due to product modalities or conditions (European Medicines Agency EMA and, 2018), it is also an option to request a product-specific waiver independent of the lists published by an individual application.

Both the FDA and the EMA published templates to be used for the application of an iPSP, a PIP, its deferral, or a waiver.

**FIGURE 1:**
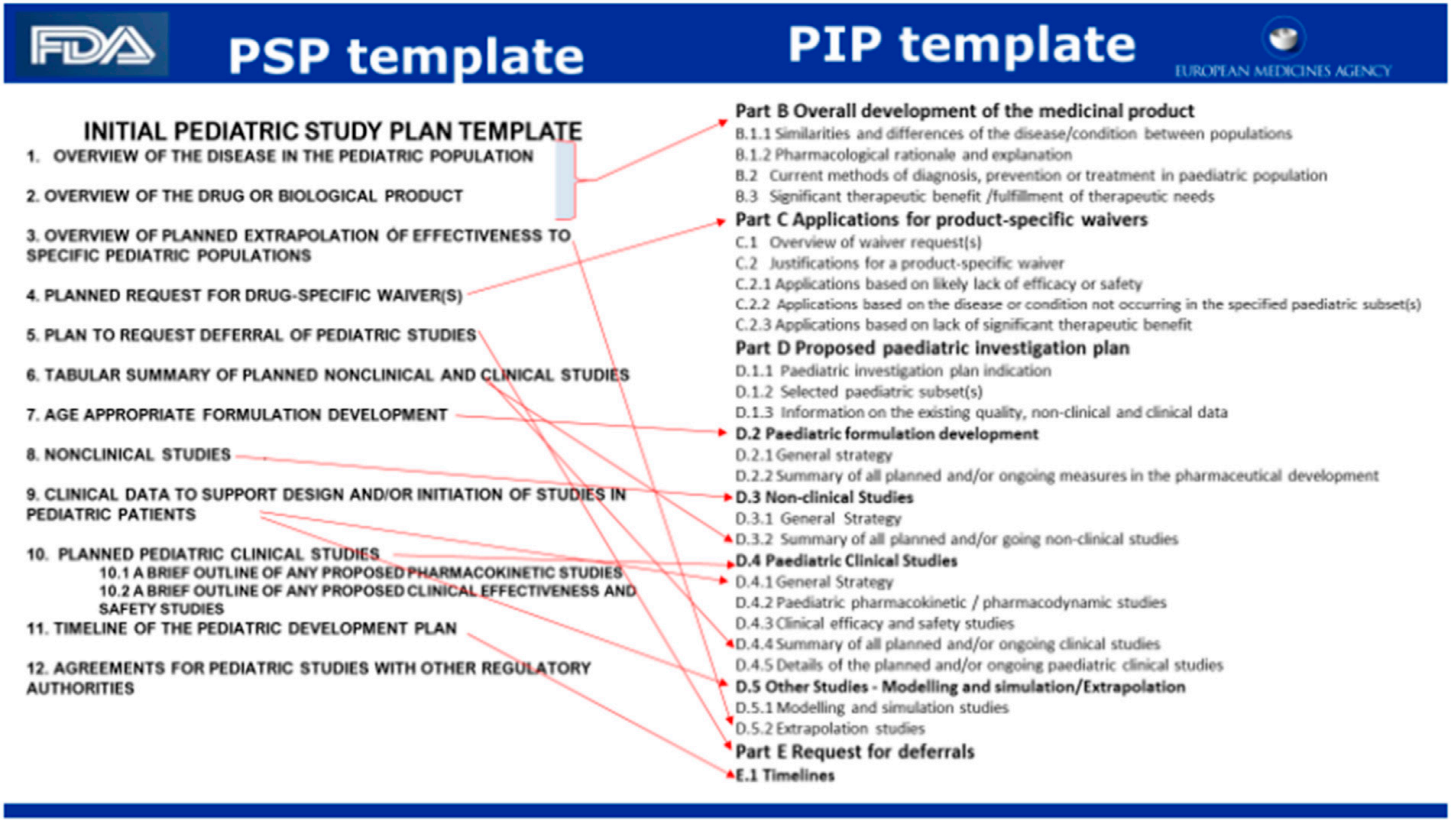
To be found: FDA/EMA Common Commentary on Submitting an initial Pediatric Study Plan (iPSP) and a Paediatric Investigation Plan (PIP) for the Prevention and Treatment of COVID-19 (FDA/EMA and Common Commentary, 2019).

Cross-references are given in Figure 1 comparison of PSP and PIP template.

### 4.4 Procedural aspects

The procedures for agreement on the content for either the PIP or the (i)PSP have some similarities but also some fundamental differences. The similarities are seen in the fact that specific scientific committees assess the submitted information. Both agencies have implemented committees to take special care of all questions, topics, and issues related to the development and approval of medicinal products for children.

One of the major differences lies in the requirements for the submission of the PIP/iPSP in the overall development programme for the medicinal product.

#### 4.4.1 Scientific committees

##### 4.4.1.1 FDA/Pediatric Review Committee

The FDA states:

PeRC is an internal committee established under the Food and Drug Administration Amendments Act (FDAAA) to carry out activities related to BPCA and PREA.

PeCr will provide consultation on and general review of paediatric information submitted to the Agency (FDA) in paediatric plans, assessments, and studies conducted by sponsors and applicants pursuant to Sections 505A and 505B of the Federal Food, Drug and Cosmetic Act (the Act) (21 U.S.C. 355 AND 355C), as amended by the FDAAA, to help ensure quality and consistency across the agency. PeRC will also provide reviews of deferrals and waivers granted under section 505B of the Act (FDA U.S. Food & Drug The and Center for Drug Evaluation and Research, 2024).

The PeRC includes employees of the FDA, including representatives from CDER (FDA U.S.Food et al., 2024), CBER (FDA U.S.Food et al., 2024), and the Office of the Commissioner, with expertise as follows:

• Pediatrics (including representation from the Office of Pediatric Therapeutics)
• Bio-pharmacology
• Statistics
• Chemistry
• Legal issues
• Paediatric ethics
• Appropriate expertise pertaining to the product under review (e.g., expertise in child and adolescent psychiatry)
• Other individuals as designated by the Secretary.

##### 4.4.1.2 EMA/Paediatric committee

Information provided on the EMA website:

The PDCO is the European Medicines Agency’s (EMA) scientific committee responsible for activities on medicines for children and for supporting the development of such medicines in the European Union by providing scientific expertise and defining paediatric needs.

The PDCO consists of the following:

• A chair, elected by serving PDCO members;
• Five members of the Committee for Human Medicinal Products (CHMP), with their alternates. These members are nominated by the CHMP;
• One member and one alternate nominated by each EU Member State that is not represented by the members nominated by the CHMP;
• One member and an alternate nominated by each of the EEA-EFTA States;
• Three members and three alternates representing patients’ associations nominated by the European Commission;
• Three members and three alternates representing healthcare professionals nominated by the European Commission.

The committee makeup will change if/when the new pharmaceutical legislation, still in the legislative procedure, will be implemented. The PDCO will be replaced by a paediatric working group, and the overall responsibility for PIP will be attributed to the Committee for Human Medicinal Products (CHMP).

#### 4.4.2 Submission request and timelines

##### 4.4.2.1 FDA

The US legislation asked for the discussion of PSPs shortly after the end of phase 2 (EoP2) testing (FDA U.S.Food and Drug, 2024).

A fixed review/communication schedule is recommended.

**FIGURE 2:**
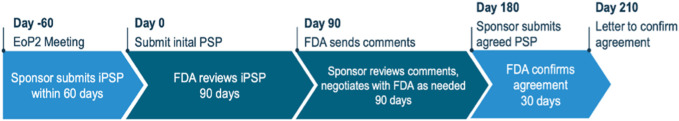
Flow chart: Dr. Max Wegner Head Regulatory Affairs/Bayer December 2022 DGRA Modul 3 (University Bonn, 2022a).

The procedure includes a validation phase and a 60-day phase for the FDA for comments. The overall procedure should be finalised by day 210. Figure 2 Flow chart gives the milestone and timelines for applicants to interact with the FDA in relation to a PSP.

##### 4.4.2.2 EMA

**FIGURE 3:**
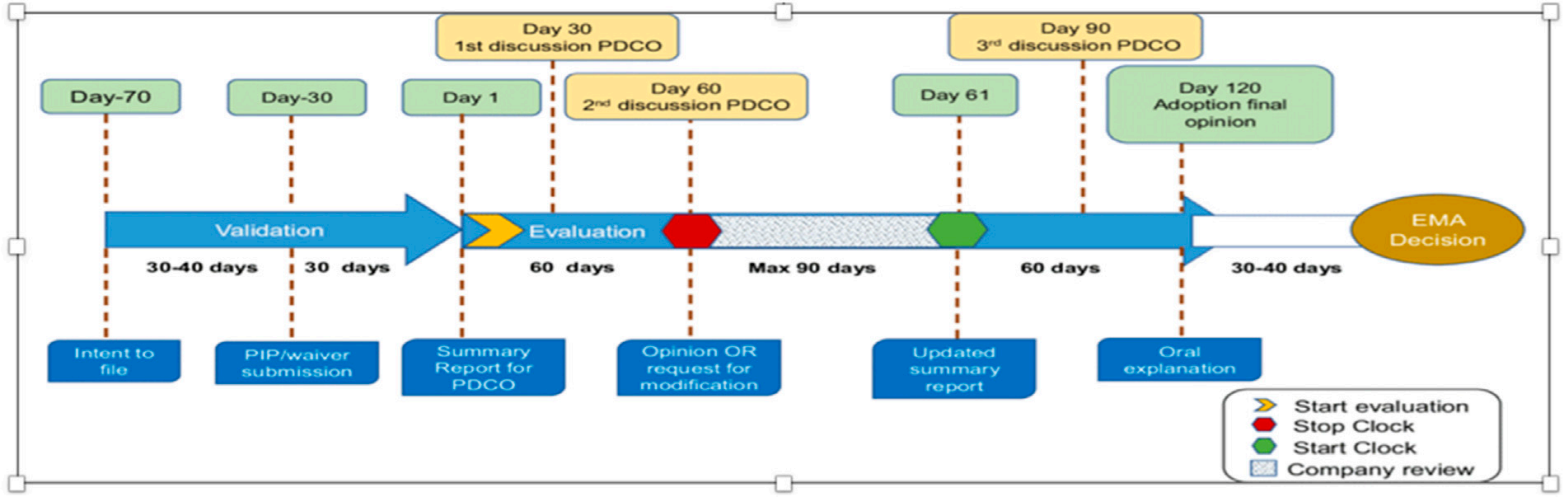
Flow chart: optimizing pediatric medicine developments in the European Union Through Pragmatic Approaches Winona Rei Bolislis et al. VOLUME 110 NUMBER 4 | October 2021 | www.cpt-journal.com (Rei et al., 2023).

The EU legislation (Article 16) requires that a PIP must be submitted after pharmacokinetic studies in adults are available. The procedure is a two-phase procedure with a stop clock to allow the applicant to answer questions raised by the PDCO before a final opinion, which will be taken by day 120. The decision will be implemented by day 150. Figure 3 Flow chart gives the milestone and timelines for applicants to interact with the EMA in relation to a PIP.

##### 4.4.2.3 Guidance for the stepwise PIP pilot

According to the EMA website:

In principle, the PIP shall be submitted no later than upon completion of the human pharmacokinetic studies in adults, that is, early in the product development and, therefore, as it is a plan, it may be subject to subsequent change as more evidence becomes available.

There might be exceptional cases where crucial information needed to define relevant parts of the plan (e.g., whether a clinical study for a whole age group is necessary, and if so, the details of the study) are not yet available to sufficiently define the key elements of the planned measure at the time of the initial PIP application. In such rare cases, where uncertainties of such a level exist, agreeing on elements in PIP studies should be avoided. It is proposed to test through a pilot the concept of the ‘stepwise PIP’ (sPIP) consisting of only a partial development programme, conditional on the development of a full PIP once the crucial information has become available.

##### 4.4.2.4 Overview of the different legal requirements in US and EU

**FIGURE 4:**
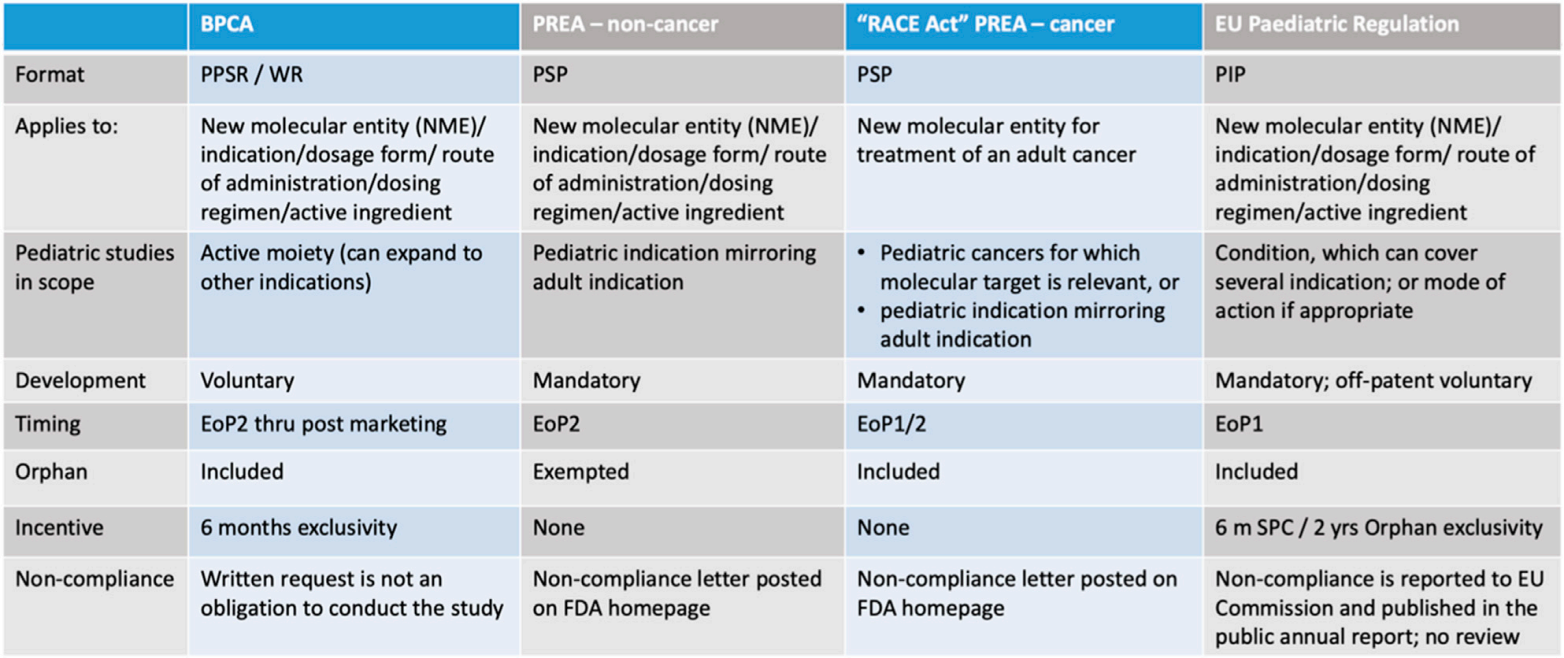
Dr. Max Wegner, Head Regulatory Affairs/Bayer, December 2022, DGRA modul 3 (University Bonn, 2022b).

Figure 4 Summary comparison table legislation US and EU.

## 5 Cooperation between the FDA and the EMA about paediatric medicinal product development

Information from the EMA website:

EMA paediatric investigational plans (PIPs), as well as general topics related to paediatric drug development (methodology, study design, endpoints, safety issues, *etc.*), are discussed in detail and documents, such as Paediatric Committee summary reports, written requests, waivers, and deferrals and, from the FDA side, new drug approval letters, Pediatric Review Committee discussions, Pediatric Research Equity Act requirements, and outcomes of workshops and working groups are shared (European Medicines Agency EMA and, 2008).

Common issues may be requested for discussion by the respective agency (EMA/PDCO and FDA) concerning paediatric oncology development plans (Paediatric Investigation Plan [PIPs] and initial pediatric study plans [iPSPs]) (European Medicines Agency EMA and, 2020b).

In addition to the *ad hoc* initiative in relation to COVID-19 medicines for children and adolescents (European Medicines Agency EMA and, 2020c), another bilateral initiative for the paediatric population focuses on medicines for cancer.

Joint guidance is available from the EMA and the FDA for medicine developers submitting a PIP to the EMA and an iPSP to the FDA on the use of a cancer medicine in children:

The purpose is to speed up the development and authorisation of cancer medicines for children, given the rarity of childhood cancers.

The guidance describes the information the two regulators typically require for their evaluations to support:

• Simultaneous submission to both regulators;
• Coordinated discussions between the regulators in the context of ‘cluster activities’ on paediatric medicines;
• International collaboration in the design and conduct of clinical trials.

The EMA and the FDA published the joint document in April 2021 (FDA U.S. Food & Drug and European Medicines Agency EMA and, 2020).

## 6 International cooperation

### 6.1 International collaboration—pediatric cluster

The following highlights the main provisions, as published by the EMA and the FDA:

In August 2007, the EMA and the FDA established monthly teleconferences between regulators called the paediatric cluster to discuss product-specific paediatric development and topics related to product classes under the terms of a confidentiality agreement. The objective of these exchanges is to enhance the science of paediatric trials and to avoid exposing children to unnecessary trials. This collaboration provides a robust ethical and scientific framework for paediatric studies. The teleconferences are held monthly, but there may be additional meetings to accommodate timelines.

Japan’s Pharmaceuticals and Medical Devices Agency (PMDA) joined these teleconferences in November 2009, and Health Canada joined in September 2010. Both agencies joined as observers and are now active participants in these monthly exchanges. Australia’s Therapeutic Goods Administration joined the teleconferences in January 2014, and they are also now an active participant in these exchanges.

The monthly discussions include ethical, safety, and paediatric study feasibility issues, as well as protocol discussions. The type of information exchanged includes the following:

• Paediatric investigation plans (PIPs);
• Written requests;
• Paediatric Committee (PDCO) discussions;
• Waivers and deferrals;
• Choice of comparator and efficacy endpoints;
• Status of ongoing paediatric studies and results of paediatric studies;
• Safety concerns, including clinical holds;
• Plans for long-term safety monitoring.

In July 2018, a new process started with the Paediatric Cluster teleconference. The FDA states: We share high-level action items with the sponsor of products following discussion at the Paediatric Cluster. The objective is to let sponsors know that their product was discussed and the agreed-upon action (FDA U.S. Food and Drug, 2024).

## 7 Information regarding the development of medicinal products for children and adolescents from some selected countries

### 7.1 The following information on the development of paediatric medicinal products was collated and summarised from relevant websites

#### 7.1.1 Japan (PMDA)

The Japanese Pharmaceuticals and Medical Devices Agency (PMDA) gives detailed information regarding their approach to improving the availability of medicinal products for children on their website.

Japan has no regulation mandating paediatric medicinal product development but has implemented several frameworks to enhance the development of drugs for children and adolescents. A series of frameworks have been discussed and were announced in 2020. These frameworks do not develop paediatric-specific laws but instead revise and extend existing regulations.

These measures include reimbursement options and an extension of the re-examination period to 10 years.

A council dedicated to unapproved and off-label drugs with a high medical need was installed.

The PDMA encouraged consideration of the clinical evaluation of drugs in paediatric patients from 10 years of age or that patients 12 years of age and older be included in clinical trials with adults.

A specific Use Drug Designation System is available (Michiyo, 2011): a system to designate drugs addressing significant unmet medical needs, such as drugs with no indication for paediatric patients, as ‘specific use drugs’ shall be defined by law, and the designated products shall be clearly qualified by law to be a candidate for a priority review system, *etc.* Criteria for such designation include the following:

1. Being used for the treatment of specific diseases;
2. Being used to treat paediatric diseases, but dosage and administration for children are not stipulated;
3. The need for specific use drugs is significantly unmet;
4. Markedly useful in that specific use.

The PMDA recommends the ICH-E11 and ICH E11 (R1) approach and is a member of the Collaboration for Paediatric Medicinal Products cluster (PMDA, 2023a).

#### 7.1.2 Canada (Health Canada)

With the institution Health Canada (the federal department responsible for helping Canadians maintain and improve their health while respecting individual choices and circumstances), Canada has one of the most highly developed concepts for the development of medicines for children and adolescents.

From the Health Canada website:

Health Canada has incentive provisions for paediatric studies.

– Six months extension of data protection under Food and Drug Regulations;
– No specific requirements to conduct paediatric studies under current Food and Drug Regulations;
– No PIP/PSP equivalent in Canada;
• Considering its stewardship role in both protecting Canadians and facilitating the provision of products vital to their health and wellbeing, Health Canada recognizes the importance of developing safe and effective medicines specifically for children;
• Applying clinically and scientifically sound methodologies to the conduct of studies is expected to provide the evidence necessary to ensure that this important patient group has access to the full benefits of therapies available to adults.

##### 7.1.2.1 Pediatric drug action plan

Health Canada’s Pediatric Drug Action Plan was developed in 2020 after extensive review and consultations with key stakeholders. The action plan will help ensure that children and youth in Canada have access not only to the medicines they need but also to age-appropriate formulations.

As part of the action plan, CPPIC is working with other government departments, as well as external partners and stakeholders, to accomplish three goals:

• Improve access to paediatric medicines and formulations;
• Increase the development of paediatric medicines and formulations;
• Provide more information to people in Canada on paediatric activities and data;

Several key measures to support the action plan:

• Modernize regulations to require drug manufacturers to provide Health Canada with meaningful information about the safety and effectiveness of drugs in children and youth;
• Develop a National Priority List of Pediatric Drugs (priority list) that are available elsewhere and needed in Canada;
• Identify the regulatory pathways and flexibilities that can be implemented to encourage industry to bring these products to Canada (Government of Canada, 2020).

The consultation on ‘Draft guidance document **on** submitting paediatric studies **and** paediatric development plans’ ran from 27 June 2023 to 26 August 2023.

Current status: Closed

#### 7.1.3 Australia (TGA)

The Therapeutics Goods Administration (TGA) is Australia’s government authority responsible for evaluating, assessing, and monitoring products that are defined as therapeutic goods. It regulates medicines, medical devices, and biologicals to help Australians stay healthy and safe.

The Australian website only gives recommendations regarding any information or data for medicinal products for children in relation to the submission of a marketing authorisation application.

The TGA references the Common Technical Document (ICH CTD) and the Module 1—regional requirements:

Advice given:

Complete the Paediatric development program form and include it in Module 1.10 of the dossier.

The form includes advice as to whether there is a paediatric development program for a medicine and provides TGA with information relevant to the Australian application about the data submitted, paediatric clinical study commitments given, and waivers received in the European Union and the United States.

The TGA has adopted internationally recognised ICH/European guidelines concerning paediatric data generation and facilitating the extrapolation of data from one patient population to another (Australian Government, 2020).

Sponsors should consider whether their medicines are likely to be used in children, and sponsors with paediatric data and appropriate formulations are encouraged to apply for registration of these medicines.

For major applications (see CTD Module 1.12):

• Complete the form Module 1.12 Paediatric Development Program)
• Include the form in the dossier.

If your registered medicine is likely to be used in children, **discuss with the TGA** how to

• Make paediatric formulations available
• Update the PI document with appropriate information on paediatric use.

The following mechanisms are available to encourage the submission of paediatric data packages:

• The Orphan Drug Program, where registration fees may be waived in conditions with a prevalence of intended users of less than 2,000 per year
• Literature-based submission to facilitate submission of modified packages based on existing published data (Australian Government, 2024).

#### 7.1.4 Brazil (ANVISA)

The competent authority for approval of medicinal products for the Brazilian market, Agencia National de Vigilancia Sanitaria (ANVISA), does not focus especially on the paediatric population but makes reference to this specific population in the list of priorities for the evaluation of medicinal product applications.

ANVISA’s RDC No. 204/2017 sets forth priorities according to specific criteria, such as whether

• The medicinal product is used for neglected, emerging, or re-emerging diseases, public health emergencies, or serious debilitating conditions where no therapeutic alternative is available or where there is a significant improvement in safety, efficacy, or adherence to treatment;
• The medicinal product is a new drug in the Brazilian market, a new pharmaceutical form, a new therapeutic indication, or a new concentration intended for the paediatric market;
• The medicinal product is a hyperimmune vaccine or serum that is to be incorporated into the National Immunisation Programme of the Ministry of Health;
• The medicinal product is an innovative or new medicinal product for an active pharmaceutical ingredient (API) manufactured in the country;
• The medicinal product is included in the list of strategic products within the scope of the Unified Health System (SUS) that is the subject of a partnership for product development (PDP) by means of the complete initial submission of all documents and studies provided for in the current regulation; and
• New drugs for the treatment, diagnosis, or prevention of rare diseases are prioritised according to ANVISA’s RDC No.205/2017 (Silva Junior, 2017).

#### 7.1.5 Switzerland (Swissmedic)

Swissmedic, the competent authority of Switzerland for the marketing authorisation and monitoring of medicinal products, gives detailed information on its website regarding its support for the development of medicines for children and adolescents, including all incentives.

Swissmedic website:

Swissmedic is the Swiss authority responsible for the authorisation and supervision of therapeutic products (Swissmedic, 2023).

In order to foster the conducting of clinical trials with children, Swissmedic offers the pharmaceutical industry—on request—an extension to the protection period for their medicinal product if it is a new development in connection with use for children.

The Ordinance on Fees levied by Swissmedic, valid as of 1 January 2013 (HgebV), foresees a fee reduction of 90% for the authorisation of, and major variations to, medicinal products with exclusively paediatric indications. Major variations include, for example, an additional indication, dosage recommendation, or dosage strength. This measure is intended to foster developments in the area of paediatric medication.

For all paediatric studies carried out in the United States or the European Union that have led to extending use to children (new indication, new pharmaceutical form, new dosage recommendation, *etc.*), Swissmedic has urged pharmaceutical companies to also submit a corresponding application to Swissmedic. Although it cannot be made mandatory under Swiss legislation on therapeutic products for authorisation holders/pharmaceutical companies to do so, they are encouraged to submit such applications voluntarily in the spirit of assuming responsibility and in the interests of the safety of medicines when treating children and adolescents.

When evaluating an authorisation application, Swissmedic checks whether the international recommendations to conduct clinical trials on paediatric populations have been taken into consideration. For that reason, Swissmedic’s Instructions for New APIS (‘Instructions, authorisation of human medicines with new active pharmaceutical ingredients and major variations’) have been updated accordingly.

#### 7.1.6 Comparison of requirements and incentives regarding the development of paediatric medicinal products in different regions/countries

Supplementary Table SA1 is provided in the annex.

## 8 Clinical trials

Marketing authorisations for medicinal products intended to treat children and adolescents are, *in principle,* based on clinical trials conducted in these populations. Conducting clinical trials in the paediatric population is one of the major obstacles to improving the current situation.

Endpoints of a clinical trial:

A good clinical trial requires a protocol with meaningful endpoints for children. Due to the expression of a disease, it might not be useful just to transfer the endpoint of the adult clinical trial to the clinical trial with a minor.

Recruitment:

The sample size of participants in a clinical trial is crucial for the outcome and acceptance of the results. Recruitment in the paediatric population means not only obtaining the agreement (informed consent) of the parents but also obtaining assent from the child involved.

Recruitment also means finding the best approach to support children and their parents during the clinical trial. The new concept of ‘decentralised clinical trials’ might be an option to overcome some hurdles in this respect.

In addition, different kinds of networks might be of relevance to encourage academia and the pharmaceutical industry to run clinical trials in children and adolescents.

Approval of a clinical trial protocol:

The approval of a clinical trial always needs the agreement given by an Ethics Committee or Institutional Review Board and a competent authority of the country in which the clinical trials take place.

### 8.1 US FDA

In the United States, *New FDA Draft Guidance Aims to Protect Children who Participate in Clinical Trials* was published in 2022 (FDA U.S. Food and Drug Administration, 2023a).

The draft guidance is intended to assist industry, sponsors and institutional review boards (IRBs) when considering the enrolment of children in clinical investigations of drugs, biological products, and medical devices.

#### 8.1.1 Ethical considerations (FDA U.S and Food and Drug Administration, 2022)

The draft guidance, *Ethical Considerations for Clinical Investigations of Medicinal Products Involving Children*, describes the ethical framework for protecting children in clinical research, including risk and benefit considerations. The draft guidance outlines and explains fundamental concepts for the ethical framework that IRBs, sponsors, and industry should consider when reviewing or conducting clinical trials involving children, including the following:

• Scientific necessity of conducting a clinical investigation in children;
• Risk categories for interventions or procedures that do not offer a prospect of direct benefit to the child;
• How to evaluate whether an intervention or procedure offers a prospect of direct benefit to the child;
• Assessment of risk for interventions or procedures with a prospect of direct benefit;
• Component analysis of the risks of interventions or procedures;
• Potential for review, under a regulatory provision, of research that is not otherwise approvable by an IRB;
• Parental or guardian permission and child assent.

### 8.2 EU/European Commission

The harmonised approach in the European Union regarding clinical trials can be found in Volume 10 of *The rules governing medicinal products in the European Union*. It contains guidance documents applying to clinical trials.

The European Clinical Trials Regulation ((EU) No 536/2014) (CTR) sets out the requirements for obtaining the approval to conduct a clinical trial.

All clinical trials in the European Union must receive the approval of a national competent authority (NCA) and a national Ethics Committee.

A number of documents in Volume 10 have been revised and updated to bring them in line with the changes required by the Clinical Trials Regulation (EU) No 536/2014. Additionally, new documents were prepared to cover new aspects introduced by the same regulation.

Recital 18 of the CTR clearly states the following: The assessment of applications for the authorisation of clinical trials should be conducted on the basis of appropriate expertise (European Commission Regulation EU, 2014).

Specific considerations for vulnerable populations are listed in Article 10 of the CTR: 1. Where the subjects are minors, specific consideration shall be given to the assessment of the application for authorisation of a clinical trial on the basis of paediatric expertise or after taking advice on clinical, ethical, and psychosocial problems in the field of paediatrics.

#### 8.2.1 Ethical consideration (European Commission: Ethical consideration for clinical trial on medicinal products conducted with minors, 2017)

Recommendations of the expert group on clinical trials for the implementation of Regulation (EU) No 536/2014 on clinical trials on medicinal products for human use are summarised in revision 1 (dated 18 September 2017) of the *Ethical considerations for clinical trials on medicinal products conducted with minors*.

The document provides recommendations on various ethical aspects of clinical trials performed with minors from birth up to the age of legal competence to provide informed consent. This will contribute to the protection of all minors who participate in clinical trials while not denying them research benefits in terms of both participation in clinical trials and access to evidence-based medicinal products.

## 9 Networks, education, and training

### 9.1 WHO Paediatric Regulatory Network (World Health Organization, 2024)

#### 9.1.1 Paediatric Regulatory Network (World Health Organization WHO, 2021a)

The WHO PRN is a global paediatric working network supporting the availability of quality medical products for children.

The network was reactivated in December 2019 as a global paediatric network supporting the availability of quality-assured medical products for children by facilitating communication, collaboration, training, and regulatory harmonisation across the development, registration, and pharmacovigilance of paediatric medical products. The network’s activities contribute efficiently to the implementation of World Health Assembly (WHA) resolutions WHA60.20 (2007) on better medicines for children, WHA69.20 (2016) on promoting innovation and access to quality, safe, efficacious, and affordable medicines for children, WHA67.20 (2014) on regulatory system strengthening for medical products, and WHA67.22 (2014) on access to essential medicines.

#### 9.1.2 WHO model list of essential medicines for children – eighth list, 2021 (World Health Organization WHO, 2021b)

Essential medicines are those that satisfy the priority healthcare needs of a population. They are selected with due regard to disease prevalence and public health relevance, evidence of efficacy and safety, and comparative cost-effectiveness. They are intended to be available in functioning health systems at all times, in appropriate dosage forms, of assured quality, and at prices individuals and health systems can afford.

The Model List is intended for use for children up to and including 12 years of age.

It compromises the need for medicinal products for children from ANAESTHETICS, PREOPERATIVE MEDICINES AND MEDICAL GASES to EAR, NOSE AND THROAT MEDICINES and dental preparations from abacavir + lamivudine to zinc sulphate.

### 9.2 US/FDA

#### 9.2.1 Pediatric trials network (Pediatric Trials, 2023)

From the overview provided on the website:

The PTN was established in 2010 as part of NICHD’s efforts for the Best Pharmaceuticals for Children Act (BPCA) program to serve as a focal point for pharmaceutical clinical trials in paediatric populations. The network, which comprises more than 100 clinical research sites across the United States, including leading paediatric hospitals and academic and research institutions, is funded through NICHD’s Obstetric and Pediatric Pharmacology and Therapeutics Branch.

The PTN’s main objectives are the following:

• Provide an environment and appropriate infrastructure for conducting safe and effective paediatric clinical trials for BPCA;
• Perform ancillary activities in support of these trials;
• Provide an atmosphere for training junior investigators in writing and conducting regulatory rigorous paediatric drug trials.

#### 9.2.2 Paediatric early-phase clinical trials network (PEP-CTN) (NIH National Cancer Institute CTEP et al., 2024)

From the information presented on the website:

The Pediatric Early-Phase Clinical Trials Network (PEP-CTN) strives to enhance NCI’s program for conducting early-phase clinical trials in children with cancer, building upon the success of the Children’s Oncology Group (COG) Phase 1 & Pilot Consortium. The overarching goal is to identify and develop effective new agents for children and adolescents with cancer through rational and efficient clinical and laboratory research.

The PEP-CTN designs and conducts paediatric early-phase trials including phase 1 trials that often include phase 2 expansion cohorts. In addition, the PEP-CTN conducts pilot studies of novel agents/regimens to determine their tolerability so that promising agents/regimens can proceed to definitive testing in phase 3 clinical trials.

Important characteristics of the PEP-CTN include the following:

• Recognition of the need for seamless transitions from phase 1 to phase 2 testing, reflected by the PEP-CTN name, emphasizing early-phase clinical trials;
• Establishment of the Pediatric Early-Phase Agent Prioritization Committee (APC) to prioritize agents for evaluation by the PEP-CTN and to expedite the pace at which novel investigational agents enter clinical testing in children with cancer;
• Addition of central monitoring for all PEP-CTN clinical trials; and
• Incorporation of relevant biological/genomic evaluations to establish eligibility for PEP-CTN clinical trials and/or to facilitate factors determining the activity of agents studied by the PEP-CTN.

#### 9.2.3 Organisations focussing on clinical trials for the paediatric population

##### 9.2.3.1 I-ACT Institute for advances clinical trials for children (I-ACT for Children’s Mission, 2023)

From the I-ACT website:

Our mission is to expedite the development and approval of safe, effective pediatric therapeutics to ensure every child has access to the treatment they need with the same urgency afforded to adults. Our work encompasses research, advocacy, education, and collaboration, all targeted towards improving pediatric treatment availability and safety standards.

##### 9.2.3.2 Critical Path Institution (Critical Path Institute, 2005)

Information provided on the C-Path website:

C-Path forms collaborative work groups comprised diverse stakeholders to identify specific barriers to developing a safe and effective therapy for a given disease and then creates tools and solutions that help drug developers overcome those barriers.

C-Path provides training for model-informed drug development (MIDD) and graduate courses (Critical Path Institute, 2024).

### 9.3 European Union/EMA

#### 9.3.1 European network of paediatric research at the European Medicines Agency (Enpr-EMA) (European Medicines Agency EMA and, 2021)

Information presented on the EMA website:

The Enpr-EMA is a network of research networks, investigators, and centres with recognised expertise in performing clinical studies in children.

Enpr-EMA enables networking and collaboration with members from within and outside the European Union, including academia and the pharmaceutical industry.

It acts as a platform for sharing good practices as well as a pan-European voice for promoting research into medicines for children.

The network does not perform clinical trials, fund studies or research, or decide on areas for paediatric research, as this is the responsibility of Member States, the European Commission, or each individual member organization.

#### 9.3.2 (EU) IMI/IHI project conect4children (c4c)

Information provided by the c4c website:

c4c is a large collaborative European network that aims to facilitate the development of new drugs and other therapies for the entire paediatric population.

It is a pioneering opportunity to build capacity for the implementation of multinational paediatric clinical trials whilst ensuring the needs of babies, children, young people, and their families are met.

c4c is committed to meeting the needs of paediatric patients thanks to a novel collaboration between the academic and the private sectors, which includes 35 academic and 10 industry partners and around 500 affiliated partners.

c4c endeavours to provide a sustainable, integrated platform for the efficient and swift delivery of high-quality clinical trials in children and young people across all conditions and phases of the drug development process.

c4c strives to bring innovative processes to all stages of clinical development by generating a new model of organisation and of the clinical development process.

By emphasizing inclusiveness and collaboration across geographical, speciality, sectoral, cultural, and societal backgrounds, it will set up a new infrastructure to support all evaluations of medicines in children.

In this manner, it will become a benchmark in the currently fragmented European clinical research environment.

Best practices and up-to-date expert advice will inform the c4c approaches and methods, which will subsequently be refined in the context of viability trials (Conect4children, 2023b).

Education within the project:

The c4c Academy Platform provides educational courses and training materials tailored to the needs of all the c4c beneficiaries involved in the conduct of paediatric clinical trials. Through these courses, c4c beneficiaries will gain greater insight and expertise into all relevant aspects of the organisation, implementation, and delivery of paediatric clinical trials, including those focused on regulatory approval of new paediatric drugs.

Accredited courses:

Advanced course on paediatric clinical trials and paediatric drug development Paediatric good clinical practice course (Conect4children, 2023a).

#### 9.3.3 Children’s medicines working group (CMWP) of the European Forum for Good Clinical Practice (European Forum and for Good Clinical Practice, 2022)

Information presented on the CMWP website:

The CMWP is a multi-stakeholder working group with its scope of activities focussing on contributing to ethical, scientific, legal, safety, and societal issues related to the design, conduct, analysis, and reporting of biomedical research and development of new medicines for children of all ages. This includes addressing the specific needs of children for child-appropriate formulations.

#### 9.3.4 European young persons advisory group network (eYPAGnet) (EYPAG Network, 2024)

Information from the eYPPAGnet website:

The European Young Persons Advisory Group Network (eYPAGnet) is a consortium of experts who facilitate meaningful patient and public involvement with children, young people, and families across Europe. eYPAGnet is renowned for setting up and working with children and young people through the forum of Young People’s Advisory Groups (YPAGs) across Europe. It was founded by the following groups: GenerationR (Liverpool, England), Kids Barcelona, Kids France, and ScotCRN (Scotland).

## 10 Challenges in the current situation and a brief outlook

The development of medicinal products for children and adolescents has, over the past years, received more attention due to the legal requirements set out in different regions. Continuous advances in physiology, pharmacology, genetics, developmental psychology, and regulatory science, including statistics, have had an influence. Treatment options have been increased but are still not at a point where optimal care for children is reached. Therefore, all possibilities for improving the medicinal treatment options for the paediatric population should be taken into account and pursued. Not the least, adequate education and training of all involved stakeholders, from the paediatric population and, where applicable, their caretakers to healthcare professionals, industry, regulators, and legislators, should be proactively undertaken.

In practice, this means that education and training considering the paediatric population should not end at the technicalities of the development of a medicine but must, by necessity, also include the specific area (i.e., paediatrics) of the regulatory field, which itself undergoes constant change following the above-mentioned advances and progress.

It follows that the harmonisation of requirements should be one important focus of activities. Other topics that should not be neglected are given here:

### 10.1 Recruitment

The recruitment of children and adolescents in clinical trials is still the most difficult topic in the process. In addition to all initiatives like Enpr-EMA and PTN, the challenges of attracting a meaningful and statistically relevant number of children (and parents) to participate in clinical trials require a substantial effort from all sides.

The concept of decentralised clinical trials might be a good step forward to circumvent that hurdle.

### 10.2 Mutual recognition

Although the concept of mutual recognition of clinical trials has recently improved, further progress is needed to reduce the number of clinical trials in the vulnerable paediatric population and bring it in line with the overall concept as set out in the European legislation: ‘unnecessary studies shall be avoided’.

### 10.3 Extrapolation

The concept of extrapolation of data gained from adult patients to adolescents or children should be followed up.

In the consultation phase of the ICH guideline E11A on paediatric extrapolation, Step 2b (EMA/CHMP/ICH/205218/2022), a total of 78 pages of comments, was received. Step 4 was expected in January 2024 (International Council of Harmonisation et al., 2024b). The implementation of Step 5 might be in 2025 (International Council of Harmonization ICH and, 2024).
