# Supplementary material for: Reflections on the regulatory field covering the development of paediatric medicinal products: a brief overview of current status and challenges
Source: Front Pharmacol. 2024 Jun 3;15:1375988. doi: 10.3389/fphar.2024.1375988 (PMC11180977; doi:10.3389/fphar.2024.1375988)
Supplement: Supplementary file 1 [file Table1.docx]

**Table 1: Comparison of requirements and incentives regarding the development of paediatric medicinal products of different regions/countries**

| **Country/Region** | **Paed Drug Dev. Mandatory** | **Scope** | **Orphan** | **Fee reduction** | **Biosimilars**  **exempted** | **Med Products exempted** | **Incentives**  **CT/MA Approval time or**  **Others like data or patents protection** | **CT - all age groups/adolescents in adults** | **Extrapolation** |
| --- | --- | --- | --- | --- | --- | --- | --- | --- | --- |
| EU | x | Indication/condition in adults | X | X | X | Homeopathic, generic, hybrid, well-established use, traditional herbal | x | x | x |
| USA | x | Adults indication | Only in oncology | X |  | Generic and dietary supplements (including herbal products regulated as  dietary supplements | x | x | x |
| Canada |  |  |  |  |  |  | x | x | x |
| Australia |  |  |  |  |  |  |  |  |  |
| Japan |  |  |  |  |  |  | x | x | x |
| Brazil |  |  |  |  |  |  | x |  |  |
| Switzerland |  |  |  | X |  |  | X |  |  |
