# Supplementary material for: Reflections on the regulatory field covering the development of paediatric medicinal products: a brief overview of current status and challenges
Source: Front Pharmacol. 2024 Jun 3;15:1375988. doi: 10.3389/fphar.2024.1375988 (PMC11180977; doi:10.3389/fphar.2024.1375988)
Supplement: Supplementary file 2 [file Presentation1.pdf]

FDA / EMA Common Commentary on Submitting an initial Pediatric Study Plan (iPSP) and Paediatric Investigation Plan (PIP) for the Prevention and Treatment of COVID-19

Given the global public health crisis resulting from the coronavirus disease 2019 (COVID-19) pandemic, FDA and EMA are providing procedural assistance to sponsors and applicants who anticipate submission of pediatric product development plans for new drugs and biological products for the treatment or prevention of COVID-19. FDA and EMA are issuing this Common Commentary to streamline administrative processes and facilitate efficient submission of an initial Pediatric Study Plan (iPSP) and Paediatric Investigation Plan (PIP). This Common Commentary addresses only the submission of an iPSP and PIP for a drug or biological product for treatment or prevention of COVID-19.

Although there are many similarities between FDA's iPSP template[1] and EMA's PIP template[2], as depicted in the diagram below, a single template would not serve the regulatory needs of both Agencies. References to existing FDA and EMA guidance on submission of an iPSP and PIP are provided at the end of this document.

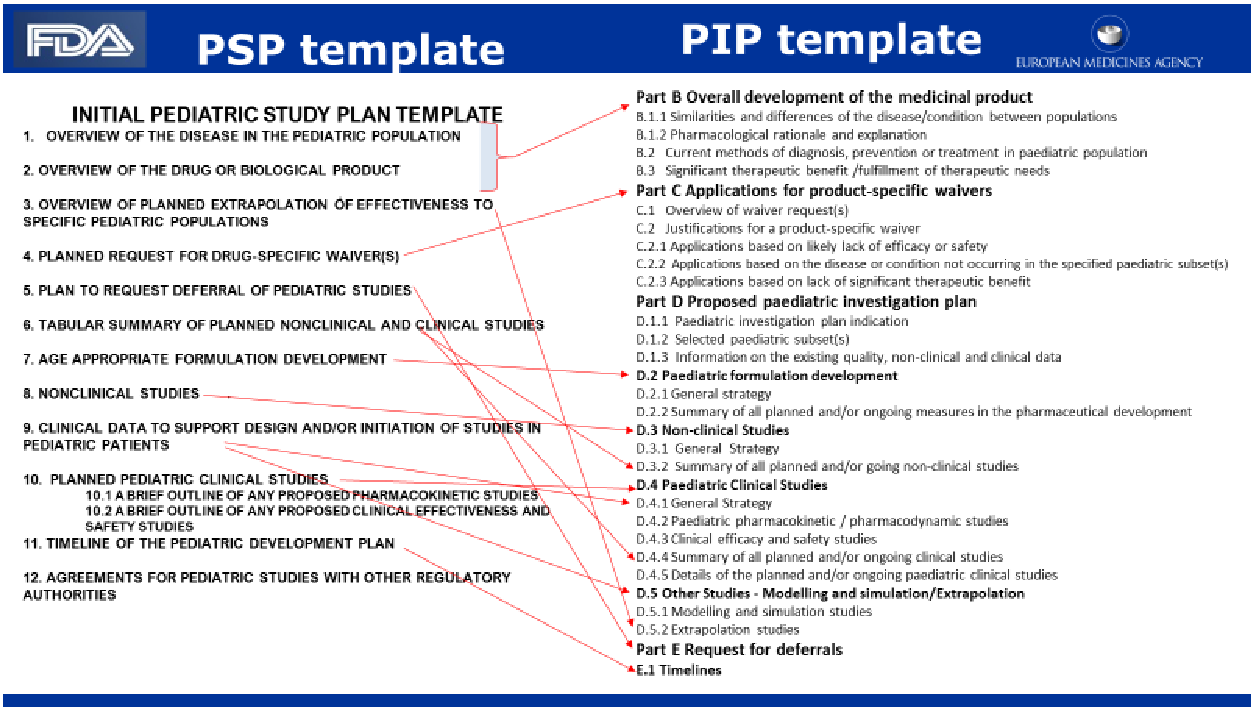

[1] The iPSP template is included in FDA's draft guidance for industry, *Pediatric Study Plans: Content of and Process for Submitting Initial Pediatric Study Plans and Amended Initial Pediatric Study Plans*, which when finalized will represent FDA's current thinking on the topic.

[2] https://www.ema.europa.eu/en/documents/template-form/template-scientific-document-part-b-f_en.doc

The table below provides a brief description of information that is often included in each section of the iPSP and the PIP. As shown, there is substantial overlap in sections of the iPSP and PIP. .

	**iPSP (20-25 pages total)**	**PIP (20-25 pages total)**<br>Some sections of the PIP template can be used in a simplified way as described below.
Overview Of The Disease In The Pediatric Population<br><br>Overview Of The Drug Or Biological Product	Section 1: Brief overview of the disease in the pediatric population (1-2 pages)<br>Section 2: Brief overview of the drug/biological product (1-2 pages)	Part B1: Short overview on the disease, the medicinal product and the pharmacological rationale (can be based on publication).<br>Focus should be on<br>- most recent research findings related to COVID-19 in relation to the pharmacological rationale of the IMP (entry into cells, binding receptors, virulence, shedding, etc.)<br>- (dis)similarity of disease/severity between adults and various paediatric age subsets as basis for potential extrapolation in view of their target population and mode of action of medicinal product<br>Part B2: Very short overview on current treatment.<br>No need to fill in Part B.3
Overview Of Planned Extrapolation Of Effectiveness To Specific Pediatric Populations	Section 3: Discussion regarding any planned use of extrapolation to support effectiveness of the product in the pediatric population (1-2 pages).	This aspect should be discussed in part D 5.2
Planned Request For Drug-Specific Waiver(s)	Section 4: Discussion of plans to request a waiver; (< 1 page).	Part C:<br>This can be concise with only the age, the grounds and arguments to support the grounds for the waiver included in the general paragraph with no need to complete C2.1, C2.2, C.2.3
Plan To Request Deferral Of Pediatric Studies	Section 5: Discussion of plans to request deferral; (< 1 page).	Part E: This can be concise with only the age, the grounds and arguments to support the grounds for the deferral included in the general paragraph.
Tabular Summary Of Planned Nonclinical and Clinical Studies	Section 6: A tabular summary that includes age groups, type of study, any comments regarding the study, and whether a request for deferral of each study is planned.	Part D.4.2: Overall summary table of all planned and/or ongoing clinical studies: Type of study/design features, Study population, dosage regimen, primary endpoint
Age Appropriate Formulation Development	Section 7: A tabular summary of the quantitative composition of the final drug product including all active and inactive ingredients, and a brief description of any plans for pediatric formulation development.	Provide all quality related information in Part D.2 (No need to fill in section D.1.3)
Nonclinical Studies	Section 8: A brief summary of the relevant nonclinical data to support studies in applicable pediatric age groups; if available, this generally includes a brief summary of pre- and post-natal studies (1-2 pages).	Brief summary of all non-clinical (existing and planned) related information in Part D.3 (No need to fill in section D.1.3)
Clinical Data To Support Design And/Or Initiation Of Studies In Pediatric Patients	Section 9: A brief summary of the top-line effectiveness and safety data in adult clinical trials as well as any PK or exposure response data that may be relevant to pediatric use. This section also typically includes plans for modeling, if any, to select the dose(s) to be studied in the pediatric trial (1-3 pages)	Part D.4 simplified with the following modified structure:<br>D.4.1: Existing adult data (PK/exposure-response, efficacy, safety) – no need to fill in part D.1.3<br>D.4.2: Tabular Summary of planned clinical trials in paediatric patients (same as section 6 in PSP)<br>D.4.3: Outline and justification for design of any proposed paediatric PK studies.<br>D.4.4: Outline and justification for design of proposed paediatric efficacy and safety studies<br>D.5.1: Plans for modeling to select doses to be studied in the paediatric trial<br>D.5.2: Use of extrapolation to support efficacy of the product in the paediatric population
Planned Pediatric Clinical Studies	Section 10.1: A brief outline of any proposed pharmacokinetic studies.<br>Section 10.2: A brief outline of any proposed clinical effectiveness and safety studies (1-5 pages)	D.4.3 - as above<br><br>D.4.4 – as above

Timeline Of The Pediatric Development Plan	Section 11: A general timeline for study completion; the timeline is often formatted as follows:<br><br>Estimated protocol submission date: No later than__________(month/year)<br>Estimated study initiation date: No later than__________(month/year)<br>Estimated study completion date: No later than__________(month/year)<br>Estimated final report submission date: No later than_________(month/year)	Part E.1<br>Provide date of initiation and completion of proposed quality, non-clinical and paediatric studies, as well as deferral requested (N/Y)
Agreements For Pediatric Studies With Other Regulatory Authorities	Section 12: A brief summary of the most recent agreed pediatric investigation plan with other regulatory authorities, if any (< 1 page)	To be submitted as supporting information:<br>• Copy of Advice/Opinion/Decision given by competent authorities of third countries<br>• Copy of FDA's Written Request

Given the need to generate clinical trial data to inform safe and effective use of products to treat and prevent COVID-19 in pediatric patients, FDA and EMA encourage early submission of an iPSP and PIP. Furthermore, FDA and EMA meet as needed to exchange information to facilitate product development for the pediatric population.

References:

FDA Draft Guidance: Pediatric Study Plans: Content of and Process for Submitting Initial Pediatric Study Plans and Amended Initial Pediatric Study Plans Guidance for Industry; https://www.fda.gov/regulatory-information/search-fda-guidance-documents/pediatric-study-plans-content-and-process-submitting-initial-pediatric-study-plans-and-amended. When finalized, this guidance will reflect FDA's current thinking on these subjects.

EMA Paediatric Investigation Plans; https://www.ema.europa.eu/en/human-regulatory/research-development/paediatric-medicines/paediatric-investigation-plans
